# Resting-state functional connectivity in adults with 47,XXX: a 7 Tesla MRI study

**DOI:** 10.1093/cercor/bhac410

**Published:** 2022-10-18

**Authors:** Chaira Serrarens, Sriranga Kashyap, Laura Riveiro-Lago, Maarten Otter, Bea C M Campforts, Constance T R M Stumpel, Henk Jansma, David E J Linden, Thérèse A M J van Amelsvoort, Claudia Vingerhoets

**Affiliations:** Department of Psychiatry and Neuropsychology, School for Mental Health and Neuroscience, Maastricht University, Maastricht, 6200 MD, The Netherlands; Department of Cognitive Neuroscience, Faculty of Psychology and Neuroscience, Maastricht University, Maastricht, 6229 EV, The Netherlands; Techna Institute, University Health Network, Toronto, M5G 2C4, Canada; Department of Psychiatry and Neuropsychology, School for Mental Health and Neuroscience, Maastricht University, Maastricht, 6200 MD, The Netherlands; Department of Psychiatry and Neuropsychology, School for Mental Health and Neuroscience, Maastricht University, Maastricht, 6200 MD, The Netherlands; Medical Department, SIZA, Arnhem, 6800 AM, The Netherlands; Department of Community Mental Health in Mild Intellectual Disabilities, Trajectum, Zutphen, 7202 AG, The Netherlands; Department of Psychiatry and Neuropsychology, School for Mental Health and Neuroscience, Maastricht University, Maastricht, 6200 MD, The Netherlands; Department of Clinical Genetics and School for Oncology and Developmental Biology, Maastricht University Medical Centre, Maastricht, 6229 ER, The Netherlands; Department of Cognitive Neuroscience, Faculty of Psychology and Neuroscience, Maastricht University, Maastricht, 6229 EV, The Netherlands; Department of Psychiatry and Neuropsychology, School for Mental Health and Neuroscience, Maastricht University, Maastricht, 6200 MD, The Netherlands; Department of Psychiatry and Neuropsychology, School for Mental Health and Neuroscience, Maastricht University, Maastricht, 6200 MD, The Netherlands; Department of Psychiatry and Neuropsychology, School for Mental Health and Neuroscience, Maastricht University, Maastricht, 6200 MD, The Netherlands; ‘s Heeren Loo Zorggroep, Amersfoort, 3818 LA, The Netherlands

**Keywords:** 47,XXX, 7 T, adults, functional connectivity, resting-state functional MRI

## Abstract

Triple X syndrome is a sex chromosomal aneuploidy characterized by the presence of a supernumerary X chromosome, resulting in a karyotype of 47,XXX in affected females. It has been associated with a variable cognitive, behavioral, and psychiatric phenotype, but little is known about its effects on brain function. We therefore conducted 7 T resting-state functional magnetic resonance imaging and compared data of 19 adult individuals with 47,XXX and 21 age-matched healthy control women using independent component analysis and dual regression. Additionally, we examined potential relationships between social cognition and social functioning scores, and IQ, and mean functional connectivity values. The 47,XXX group showed significantly increased functional connectivity of the fronto-parietal resting-state network with the right postcentral gyrus. Resting-state functional connectivity (rsFC) variability was not associated with IQ and social cognition and social functioning deficits in the participants with 47,XXX. We thus observed an effect of a supernumerary X chromosome in adult women on fronto-parietal rsFC. These findings provide additional insight into the role of the X chromosome on functional connectivity of the brain. Further research is needed to understand the clinical implications of altered rsFC in 47,XXX.

## Introduction

Triple X syndrome is a sex chromosomal aneuploidy (SCA) characterized by the presence of a supernumerary X chromosome, resulting in a karyotype of 47,XXX in affected females. 47,XXX has an estimated incidence of about one in 1,000 female newborns (Otter et al. 2010). Individuals with 47,XXX display a variable, but generally mild, phenotype and it is hypothesized that the phenotypic traits result from overexpression of genes on the X chromosome that escape X-inactivation (Nielsen et al. 2020). Therefore, it is estimated that only 16% of cases are clinically diagnosed (Viuff et al. 2015). However, deficits in children and adolescents with 47,XXX have been found in several domains, including motor development, speech and language, educational achievement, and interpersonal relationships (Leggett et al. 2010; Lenroot et al. 2014; Urbanus et al. 2021). In addition, increased rates of neurodevelopmental disorders including autism spectrum disorder (ASD), and attention deficit hyperactivity disorder (ADHD) have been reported (Van Rijn 2019). Furthermore, anxiety, depressive, and psychotic disorders are more prevalent in individuals with 47,XXX compared to the general population (Otter et al. 2010; Green et al. 2019). On a cognitive level, mild learning disabilities and executive functioning deficits have been described in children and adolescents with 47,XXX (Tartaglia et al. 2010; van Rijn and Swaab 2015; van Rijn et al. 2018). Individuals with 47,XXX have a lower mean full-scale IQ (FSIQ), with the IQ normal curve shifted to the left compared to healthy controls, with verbal IQ (VIQ) more impaired compared to performance IQ (PIQ;Otter et al. 2010; Tartaglia et al. 2010). Finally, social functioning and social cognition deficits have been described in children (Lee et al. 2012; van Rijn et al. 2014; Wilson et al. 2019), as well as in adults with 47,XXX (Otter et al. 2021).

Several neuroimaging studies have revealed structural brain abnormalities in 47,XXX including alterations of subcortical volumes, cortical thickness and surface area, and cortical folding in children and adolescents (Patwardhan et al. 2002; Lenroot et al. 2014; Raznahan et al. 2016; Reardon et al. 2016; Fish et al. 2017; Nadig et al. 2018). In adulthood, alterations in subcortical and lateral ventricle volumes, and cortical surface area have been found (Serrarens et al. 2022). However, (functional) connectivity of the brain in 47,XXX has not been investigated. Resting-state functional magnetic resonance imaging (rs-fMRI) can be used to measure brain activity when subjects are at rest, i.e. in the absence of an experimental task or stimulation, allowing for measures of temporal correlation of the blood oxygen level dependent (BOLD) signal among spatially distributed brain regions (functional connectivity). In contrast to other SCAs, including Turner syndrome (45,X) and Klinefelter syndrome (47,XXY), to the best of our knowledge, there have been no rs-fMRI studies conducted in 47,XXX. Therefore, it remains unclear whether resting-state functional connectivity (rsFC) is affected in females with a supernumerary X chromosome. Understanding how rsFC of the brain is affected in 47,XXX may provide insight into the neural mechanisms underlying X chromosome-linked cognitive, behavioral and psychiatric phenotypes in healthy and affected individuals. A recent rs-fMRI study comparing individuals with Klinefelter syndrome with male healthy controls has shown increased rsFC strength in the left dorsolateral prefrontal cortex (DLPFC) in 47,XXY—reflecting fronto-parietal network functional hyperconnectivity with rsFC networks including visual/cerebellum, default-mode/limbic, ventral attention/somatomotor and dorsal attention/frontoparietal (Whitman et al. 2021). In contrast, a rs-fMRI study in individuals with Turner syndrome showed reduced rsFC strength in fronto-parietal and dorsal attention networks in 45,X compared to control women (Green et al. 2018). In addition, they showed reduced rsFC between the intraparietal sulcus (IPS) and frontal and cerebellar regions. Finally, another rs-fMRI study showed reduced rsFC strength within the postcentral gyrus/IPS, angular gyrus, cuneus and the cerebellum in girls with Turner syndrome compared to matched controls (Xie et al. 2017). This study also showed reduced rsFC between these seed regions and target regions. Although altered rsFC has been demonstrated in other SCAs, it remains unclear whether rsFC is affected in women with a supernumerary X chromosome.

Therefore, the aim of the present study was to compare functional connectivity at rest between adult individuals with 47,XXX and sex-matched healthy controls using ultra-high field (7 Tesla) resting-state functional magnetic resonance imaging. Furthermore, given previous evidence of lower IQ, ﻿impaired social functioning and social cognition, and executive functioning deficits in 47,XXX, we examined whether the variability of these cognitive parameters was related to variability in rsFC in 47,XXX.

## Materials and methods

All procedures in this study were performed in accordance with the ethical standards established by the respective national and institutional committees regarding human experimentation and in accordance with the Declaration of Helsinki. In addition, all procedures involving human subjects were approved by the Medical Ethics Committee of the Maastricht University Medical Centre, Maastricht, the Netherlands (METC143051/NL46871.068.14). Written informed consent was obtained from all participants.

### Participants

Nineteen adults with 47,XXX and 21 age- and sex-matched healthy controls, aged 18–59, were included in this study. The Dutch (NL) and Flemish (B) individuals with 47,XXX were recruited through the 47,XXX support group, clinicians, clinical geneticists, pediatricians and gynecologists. Healthy controls were recruited independently through local advertisement. General inclusion criteria were 1) 18 years or older of age 2) mental capacity to give informed consent and 3) a sufficient command of the Dutch language. Individuals with 47,XXX were included on the basis of a confirmed 47,XXX karyotype or a mosaic 46,XX/47,XXX karyotype with at least 85% cells with an extra X chromosome. Exclusion criteria for all study participants were 1) being under legal guardianship, 2) contraindications for MRI, and 3) pregnancy.

### Instruments

A shortened version of the Dutch Wechsler Adult Intelligence Scale, Third Edition (WAIS-III; Velthorst et al. 2013) was administered to all participants to estimate the level of intellectual functioning. The Cambridge Neuropsychological Test Automated Battery (CANTAB; ﻿Cambridge Cognition, Cambridge, UK; see www.cantab.com) was used to assess multiple levels of cognitive functioning. See Table 1 for an overview of subtests and outcome measures included used to measure social cognition and executive functioning. The Dutch translation of the informant/observer version of the Social Responsiveness Scale for adults (SRS-A) was used to assess social responsiveness, which is considered a screening instrument for ASD, in all participants (Constantino et al. 2012). The SRS-A questionnaire is subdivided into 4 subscales including 1) social awareness, 2) social communication, 3) social motivation, and 4) rigidity and repetitive behavior. SRS-A scales are reported as T-scores with scores <40 indicating high social functioning, scores between 40 and 59 indicating normal social functioning, scores between 60 and 75 indicating mild to moderate social deficits and scores ≥76 indicating severe deficits. The ﻿Mini International Neuropsychiatric Interview (M.I.N.I.; Sheehan et al. 1998) was used to obtain psychiatric diagnoses, which were, however, not verified by an experienced psychiatrist or psychologist.

**Table 1:** Overview of CANTAB subtests assessed.

Domain	Subtest	Outcome measure	Direction
Social cognition	Emotion Recognition Task (ERT)	Total number of correctly identified emotions	Higher is better
Attentional set-shifting	Intra-Extra Dimensional Set Shift (IED)	Total errors in the extra dimensional stage of the task	Lower is better
Spatial planning and working memory	One Touch Stockings of Cambridge (OTS)	Total numbers of problems solved on first choice	Higher is better
Strategy and working memory	Spatial Working Memory (SWM)	Total errors	Lower is better
		Strategy	Lower is better

### MR data acquisition

MR data acquisition was carried out at Scannexus B.V. (https://scannexus.nl) on a Siemens Magnetom 7 T scanner (Siemens Healthineers, Erlangen, Germany) using a 1Tx/32Rx commercial head coil (Nova Medical Inc., Wilmington, MA, USA). Anatomical data were acquired using a 3D-MP2RAGE sequence (Marques et al. 2010); repetition time (TR) = 5,000 ms; echo time (TE) = 2.51 ms; inversion times TI1/TI2 = 900/2,750 ms; *α*_1_/*α*_2_ = 5°/3°; phase partial Fourier = 6/8; GRAPPA = 2 with 24 reference lines; bandwidth = 248 Hz/Px; nominal voxel size = 0.7 × 0.7 × 0.7 mm^3^; acquisition time = 10:57 min. Resting-state fMRI data were acquired using a gradient-echo multi-band 2D gradient-echo EPI sequence: 84 slices; TR = 1,700 ms; TE = 19 ms; *α* = 64°; SMS = 3; FOV = 198 × 198 mm^2^; voxel size = 1.5 × 1.5 × 1.5 mm^3^; partial Fourier = 6/8; 200 volumes in total and acquisition time = 6:10 min. Five volumes of opposite phase-encoded scans were acquired immediately following the resting-state run. For the resting-state scan, participants were instructed not to think about anything in particular and focus on a fixation-cross in the middle of an isoluminant gray screen.

### Preprocessing of imaging data

Preprocessing of rs-fMRI data was carried out using tools from *FSL* (FMRIB Software Library, Oxford University, UK, version 6.0; Jenkinson et al. 2012) and *ANTs* (Advanced Normalization Tools; Avants et al. 2011). First, 2 EPI volumes with opposite phase encoding directions (A> > P and P> > A) were brain extracted using *FSL BET* and registered to one another using *FSL FLIRT*. Field maps were estimated using *FSL TOPUP*. The following steps were then applied to the rs-fMRI timeseries data using *FSL*: 1) brain extraction using *BET*, 2) head motion correction using *MCFLIRT*, 3) susceptibility distortion correction using an adapted version of *FSL’s epi_reg* script using the fieldmap estimated from *TOPUP*, 4) slice time correction using *slicetimer*, and 5) spatial smoothing with *FSL SUSAN* using a Gaussian kernel full-width at half-maximum of 4.5 mm. Rigid registration between the processed rs-fMRI and T1-weighted anatomical image was carried out using *boundary-based registration (BBR)* as implemented in *FSL FLIRT*. The T1w data was registered to the 2 mm MNI152 template using SyN diffeomorphic (non-linear) transformation with parameters as implemented in the *antsRegistrationSyN.sh* script. This algorithm has been shown to provide superior performance compared to most other registration algorithms used in neuroimaging (Klein et al. 2009; Avants et al. 2011). Transformations and warps were then applied to the distortion corrected, slice time corrected and smoothed volumes to bring the rs-fMRI data into MNI space with as few interpolation steps as possible. In 4 datasets, the acquired opposite phase encoded datasets were unusable. To overcome this issue, we used the dockerized version of the *Synb0-DisCo* (Schilling et al. 2019, 2020) algorithm as an intermediate step to estimate an undistorted EPI with the contrast similar to the EPI data. This synthesized data was used instead of the opposite phase-encoded data as input to *FSL TOPUP* (pipeline identical to Fig. 1 in Schilling et al. 2020). Subsequent processing steps were the same as outlined above. Processing and registrations were carefully visually inspected at every stage to ensure quality control.

Independent Component Analysis based Automatic Removal of Motion Artifacts (*ICA-AROMA*; version 0.3 beta; Pruim et al. 2015) was then used to detect and remove motion artifacts from the individual rs-fMRI data, resulting in denoised rs-fMRI data. To facilitate the use of *ICA-AROMA*, the data were analyzed in 2 mm MNI space. White matter (WM) and cerebrospinal fluid (CSF) maps were segmented from the T1w data using *SPM12* (Ashburner and Friston 2005) and adapted in-house code (Kashyap 2021). WM and CSF tissues were transformed to the MNI152 2 mm template by applying the *ANTs* warps using the label interpolator in *antsApplyTransforms*. Using the WM and CSF maps as input, nuisance time series were generated from the *ICA-AROMA* output. A general linear model (GLM) was generated from the denoised rs-fMRI data and nuisance timeseries to obtain residual functional activity. Finally, removal of residual functional activity attributed to WM and CSF and high-pass temporal filtering (100 s) was applied to the denoised rs-fMRI data.

### Analysis of rs-fMRI data

*FSL MELODIC* was used to perform temporal concatenation of the denoised rs-fMRI data, then decomposing the data into 20 independent components (Smith et al. 2009). These 20 components were chosen as template maps because they have been shown to closely correspond to brain networks identified in thousands of individuals across a wide range of tasks (Smith et al. 2009). A spatial cross-correlation between the 20 independent components identified in our study and 10 major resting-state functional networks as identified by Smith et al. (2009) was calculated using *FSL’s fslcc* command. Resting-state networks (RSNs) that yielded a spatial correlation with a Pearson’s *r* value higher than 0.4 were included for further analysis. This procedure identified 7 RSNs that showed medium or high spatial correspondence (>0.4) with Smith’s 10 major resting-state functional networks (Table 2). We then applied *FSL’s dual regression* (Beckmann et al. 2009) to generate subject-specific spatial maps and associated time series derived from the set of spatial maps from the group-average analysis. First, the 7 independent components were regressed against the denoised rs-fMRI data, resulting in subject-specific time series. These component-specific time series were variance normalized and regressed against the same denoised rs-fMRI data, resulting in subject-specific spatial maps. Differences between groups in functional connectivity in the 7 RSNs was compared using *FSL randomise* (5,000 permutations) with a two-sample *t*-test. Statistical thresholding was performed with threshold-free cluster enhancement (TFCE; Smith and Nichols 2009) and was set at *P* < 0.05 family wise error (FWE)-corrected for multiple comparisons. Bonferroni correction (two-tailed) was applied to correct for the number of included brain networks, resulting in a reported significance threshold of *P* < 0.00357 (=0.05/(7 networks * 2 directions)).

**Table 2:** Seven RSNs identified in a sample comprising of individuals with 47,XXX and healthy controls, corresponding to 7 RSNs found by Smith et al. (2009).

Resting-state functional network	Pearson’s *r*
Medial visual network	0.782
Occipital pole visual network	0.569
Default mode network	0.699
Auditory network	0.479
Executive control network	0.547
Right fronto-parietal network	0.545
Left fronto-parietal network	0.543

### Statistical analyses

Statistical analyses were performed in *R*, version 3 (R Core Team 2013). First, differences in group demographics including age, FSIQ, VIQ, and PIQ were examined using Mann–Whitney *U* tests and independent samples *t*-tests according to the normality of data distribution. Second, cognitive outcome measure scores were transformed into standardized *Z*-scores to identify outliers. Z-scores smaller than −3 or larger than 3 were considered outliers. Outliers were not detected. Normally and non-normally distributed raw scores on the Emotion Recognition Test (ERT), Intra-Extra Dimensional Set Shift (IED), One Touch Stockings of Cambridge (OTS), and the Spatial Working Memory (SWM) test of the CANTAB were compared between groups using an independent samples *t*-test and Mann–Whitney *U* tests, respectively. Normally and non-normally distributed total SRS-A T-scores and T-scores for SRS-A subscales were compared using independent samples *t*-tests and Mann–Whitney *U* tests, respectively. In case cognitive outcome measure scores or social functioning scores differed significantly between 47,XXX subjects and healthy controls, relationships between these cognitive parameters and mean functional connectivity values (parameter estimates) extracted from the significant voxels were calculated using Pearson’s or Spearman’s rank correlation coefficients, separately for 47,XXX and healthy controls.

## Results

### Demographics

Sample demographics are presented in Table 3. There was no significant difference in age between groups. Individuals with 47,XXX had a significantly lower FSIQ, VIQ, and PIQ compared to healthy controls. Head motion was compared between individuals with 47,XXX (mean: 0.38; SD: 0.22) and healthy controls (mean: 0.41; SD: 0.42), showing no significant difference in head motion between groups (*U* = 158, *P* = 0.267).

**Table 3:** Sample demographics.

		47,XXX Mean (SD)		Healthy controls Mean (SD)	Statistic	*P*
	*N*		*N*			
Age	19	28.89 (10.90)	21	32.95 (11.99)	*U* = 239.5	0.284
FSIQ	17	87.06 (10.76)	21	99.71 (12.62)	*t* = 3.82	**0.002**
VIQ^a^	17	81.47 (13.15)	21	95.10 (13.27)	*U* = 269.5	**0.008**
PIQ^a^	17	89.71 (14.11)	21	102.86 (18.45)	*t* = 2.42	**0.021**
**Diagnosis** ^b^						
Major depressive disorder	1/19		0/21			
Dysthymic disorder	1/19		0/21			
Agoraphobia	2/19		1/21			
Social phobia	1/19		0/21			
Obsessive compulsive disorder	2/19		0/21			
Psychotic disorder	3/19		0/21			
Generalized anxiety disorder	5/19		0/21			

Numbers in bold reflect significant between group differences. FSIQ: full-scale intelligence quotient; VIQ: verbal intelligence quotient; PIQ: performance intelligence quotient.

^a^No significant difference between VIQ and PIQ in either 47,XXX or healthy controls.

^b^Measured using the M.I.N.I.

### Cognitive assessment and social functioning

Social cognition and executive functioning scores are summarized in Table 4. Individuals with 47,XXX showed significantly lower ERT scores compared to healthy controls. There were no significant differences between groups in IED, OTS, or SWM scores. SRS-A T-scores are summarized in Table 5. Individuals with 47,XXX had significantly higher scores on 3 SRS-A subscales: social awareness, social communication and social motivation, as well as on total SRS-A score. There was no significant difference between groups in score of SRS-A subscale rigidity and repetitive behavior.

### RsFC differences

**Fig. 1:**
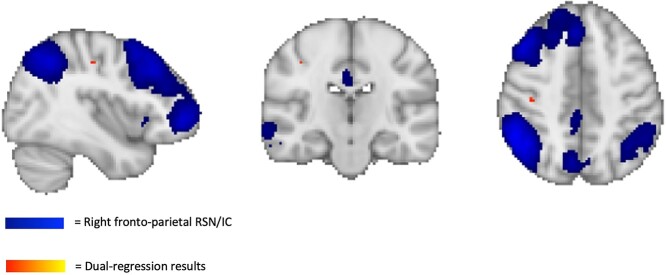
Increased rsFC of the right fronto-parietal network with the right postcentral gyrus in individuals with 47,XXX. The independent component representing the right fronto-parietal network which was used as input for the dual regression is plotted in blue. Significant voxels that were found in the right postcentral gyrus (*x* = 25, *y* = 53, *z* = 58) are plotted in red. Results shown are thresholded at *P* < 0.00357, FWE-corrected with threshold-free cluster enhancement. Results are superimposed on a MNI152 standard space template image.

We showed significantly increased rsFC of the fronto-parietal network with the right postcentral gyrus (4 voxels, location of maximum intensity voxel at x,y,z: 25, 53, 58) within the 47,XXX group. Results are shown in Figure 1. We did not observe significant group differences in any of the other networks.

### Relationship with cognition and social behavior

We found no significant correlation between rsFC connectivity of the right postcentral gyrus and FSIQ, VIQ and PIQ in either 47,XXX or healthy controls. In addition, we did not show significant correlation between social cognition and rsFC connectivity of the right postcentral gyrus in either 47,XXX or healthy control subjects. Lastly, significant correlations between SRS-A scores and rsFC of the right postcentral gyrus were not present in both groups.

## Discussion

To the best of our knowledge, this is the first MRI study investigating rsFC in adults with 47,XXX using ultra-high field 7 Tesla functional MRI. Using an ICA approach, we demonstrated significantly increased functional connectivity of the fronto-parietal network with the right postcentral gyrus in 47,XXX. However, rsFC variability was not associated with IQ and social cognition and social functioning deficits in adult individuals with 47,XXX.

The finding of altered fronto-parietal rsFC in adults with 47,XXX is in line with the literature in children and adolescents in other SCAs. Children and adolescents with Klinefelter syndrome (47,XXY) showed functional hyperconnectivity at rest between frontal and parietal regions (Whitman et al. 2021), while in children and adolescents with Turner syndrome (45,X) functional hypoconnectivity at rest between frontal and parietal regions (Xie et al. 2017; Green et al. 2018) have been reported. To the best of our knowledge, previous rsFC studies in adults with other SCAs than 47,XXX have not been performed. Together these findings are indicative of an effect of X chromosome gene-dosage on fronto-parietal rsFC. In order to investigate these X chromosome gene-dosage effects on rsFC in more depth, future studies including larger sample sizes and a more diverse group of SCAs are required. Combined, findings in different SCAs also suggests a similar X chromosome gene-dosage effect on fronto-parietal rsFC across different developmental periods. Therefore, it could be hypothesized that altered rsFC in these SCAs could be considered as a trait rather than a state characteristic. Longitudinal studies are warranted investigating rsFC in SCAs across developmental trajectories.

**Table 4:** Between group differences in cognitive functioning per included domain.

		47,XXX Mean (SD)		Healthy controls Mean (SD)	Statistic	*P*
	*N*		*N*			
**Social cognition** ERT total correct	18	105.17 (17.05)	21	119.71 (14.38)	*t* = 2.89	**0.006**
**Attentional set-shifting** IED total errors extra-dimensional stage	18	8.22 (8.57)	21	8.29 (10.18)	*U* = 171	0.618
**Planning** OTS Problems solved on first choice	19	10.68 (2.89)	21	10.71 (2.69)	*U* = 192.5	0.859
**Strategy and working memory** SWM total errors SWM strategy	19 19	11.84 (11.03) 15.00 (4.44)	21 21	7.57 (5.93) 14.24 (3.00)	*U* = 171.5 *t* = −0.63	0.455 0.532

The number in bold reflects a significant between group difference. ERT: emotion recognition task; IED: intra-extra dimensional set shift; OTS: ﻿One Touch Stockings of Cambridge; SWM: spatial working memory.

**Table 5:** Between group differences in social functioning.

		47,XXX Mean (SD)		Healthy controls Mean (SD)	Statistic	*P*
	*N*		*N*			
Social awareness score	18	58.28 (10.87)	21	49.52 (11.49)	*t* = −2.43	**0.020**
Social communication score	18	56.89 (9.96)	21	46.81 (8.00)	*U* = 82.5	**0.003**
Social motivation score	18	56.22 (8.72)	21	45.86 (7.45)	*t* = −4.01	**<0.001**
Rigidity and repetitive behavior score	18	55.33 (13.34)	21	49.24 (10.55)	*U* = 128	0.087
Social functioning total score	18	57.61 (10.47)	21	47.48 (9.68)	*U* = 82	**0.003**

Numbers in bold reflect significant between group differences.

We found significantly increased functional connectivity of the fronto-parietal network with the right postcentral gyrus in 47,XXX compared to healthy controls. The fronto-parietal RSN includes parts of the lateral prefrontal cortex and posterior parietal cortex and has been implicated in cognitive control (Zanto and Gazzaley 2013). Cognitive control refers to processes that allow information processing and behavior to vary depending on present conditions, prevailing contexts, and future plans (Nee 2021). Executive functions are a set of cognitive processes necessary for cognitive control. Alterations of fronto-parietal rsFC as reported here in 47,XXX have been associated with major idiopathic neurodevelopmental and psychiatric disorders, including ADHD, ASD, schizophrenia, and depression, in which executive functioning deficits are common (Menon 2011; Lin et al. 2015; Marek and Dosenbach 2018). Thus, it seems that there is an overlap in rsFC between the 47,XXX phenotype and idiopathic neurodevelopmental and psychiatric disorders. It might be interesting for future studies to also compare fronto-parietal rsFC between 47,XXX individuals with and without a psychiatric disorder. Unfortunately, our sample was not sufficient to elaborate on this topic.

The postcentral gyrus, which was found to show increased rsFC with the fronto-parietal network in 47,XXX, is the site of the primary somatosensory cortex and forms part of the sensorimotor RSN. This RSN corresponds closely to activations seen in motor-tasks (Biswal et al. 1995). Motor deficits have been described in 47,XXX (Leggett et al. 2010; Otter et al. 2010, 2022). However, motor abilities of 47,XXX individuals were not assessed in the current study. Therefore, the relationship between rsFC and motor deficits seen in 47,XXX needs to be further investigated.

In a previous study using a largely overlapping sample, we analyzed multiple levels of brain morphology and showed lower subcortical volumes and lower cortical surface area in the superior temporal gyrus and superior frontal gyrus of the right hemisphere in adults with 47,XXX, but no structural alterations in the postcentral gyrus, lateral prefrontal cortex and posterior parietal cortex (Serrarens et al. 2022). Interestingly, here we demonstrated altered fronto-parietal rsFC in 47,XXX adults, suggesting that alterations of functional connectivity at rest are not colocalized with alterations of brain structure in adult women with a supernumerary X chromosome. These findings suggest that altered fronto-parietal rsFC in 47,XXX is not an epiphenomenon of surface area loss.

Executive functioning problems including deficits in inhibition, mental flexibility, sustained attention and (visual) working memory have previously been reported in children and adolescents with 47,XXX (Tartaglia et al. 2010; van Rijn and Swaab 2015; van Rijn et al. 2018). However, in this study deficits in 47,XXX adults in attentional set-shifting, planning, strategy and working memory, which are considered components of executive functioning, were not shown. Absence of executive functioning deficits was also reported in the study of Otter et al. (2022), in which cognitive functioning in adults with 47,XXX was investigated, using a largely overlapping sample of 47,XXX individuals. Since components of executive functioning were not altered in 47,XXX we did not investigate their relationship with rsFC. Another study in children and adolescents with Turner syndrome also did not demonstrate correlations between the fronto-parietal RSN established with ICA and measures of executive functioning, suggesting that altered fronto-parietal rsFC not directly influences executive functioning in SCAs (Green et al. 2018). Yet, decreased functional activation in frontal and parietal regions has been shown during working memory (Haberecht et al. 2001; Hart et al. 2006; Bray et al. 2011) and response inhibition (Tamm et al. 2003) tasks in Turner syndrome. More research is necessary to investigate the contribution of rsFC to executive functioning in SCAs.

Our results also did not show an association between fronto-parietal rsFC variability and IQ, and social cognition and social functioning in 47,XXX. However, we reported significantly lower IQ and deficits in social cognition and social functioning in 47,XXX, which is in line with other studies in children, adolescents, and adults with 47,XXX (Lee et al. 2012; van Rijn et al. 2014; Wilson et al. 2019; Otter et al. 2021). More research is warranted to investigate the possible relationship between brain functional connectivity and IQ, social cognition and social functioning in adult individuals with 47,XXX.

### Strengths and limitations

Our study is the first to investigate rsFC alterations in adults with 47,XXX. Especially, our work in 47,XXX presented here add to existing studies of other SCAs such as Turner and Klinefelter syndromes shedding more insight into the X chromosome gene-dosage effect on rsFC. Another important strength of this study is the use of ultra-high field 7 T rs-fMRI data. Previous studies in 45,X and 47,XXY acquired rs-fMRI data using a conventional field strength of 3 T. The main advantage of 7 T rs-fMRI is that the increased signal-to-noise ratio (SNR) at ultra-high field allows imaging with higher spatial resolutions (Barth and Poser 2011; Uğurbil 2021). Additionally, the BOLD signal is spatially more accurate due to an increased extravascular weighting at ultra-high fields resulting in improved spatial specificity compared to lower field strengths (Uludaǧ et al. 2009). Despite the strong merits and novelty of our study, these findings should also be considered in light of certain limitations. First, our sample size was relatively small compared to previous rs-fMRI studies in other SCAs, resulting in limited power to detect statistically significant differences. The absence of a relationship between rsFC variability and IQ, social cognition, and social functioning could also be due to the relatively small sample size. A recent study has argued that large sample sizes may be necessary to reliably relate brain function to behavior (Marek et al. 2022). However, given that 47,XXX is a relatively rare genetic disorder, recruitment of large sample sizes is difficult and requires international collaborative consortia. Second, between-group findings could be related to differences in IQ, as opposed to or in addition to the group itself. Future studies could address this problem including an IQ-matched healthy control group. Third, ascertainment bias is a well-known limitation in 47,XXX studies as patients presenting more severe phenotypes are more likely to be clinically recognized and enrolled in research. Therefore, our sample may not be representative of all individuals with 47,XXX. Finally, the cross-sectional nature of our data makes it difficult to assess possible age-varying patterns of rsFC in 47,XXX individuals, stressing the need for longitudinal studies investigating rsFC in 47,XXX.

## Conclusion

In conclusion, our results suggest an effect of a supernumerary X chromosome in adult women on fronto-parietal rsFC organization. These findings provide additional insight into the role of the X chromosome on functional connectivity of the brain. Further research is needed to understand the clinical implications of altered rsFC in 47,XXX.
